# HDAC1 promoted migration and invasion binding with TCF12 by promoting EMT progress in gallbladder cancer

**DOI:** 10.18632/oncotarget.8740

**Published:** 2016-04-15

**Authors:** Junyi He, Sheng Shen, Weiqi Lu, Yuhong Zhou, Yingyong Hou, Yong Zhang, Ying Jiang, Houbao Liu, Yebo Shao

**Affiliations:** ^1^ Department of General Surgery, Zhongshan Hospital, Fudan University, Shanghai 200032, China; ^2^ Department of Clinical Oncology, Zhongshan Hospital, Fudan University, Shanghai 200032, China; ^3^ Department of Pathology, Zhongshan Hospital, Fudan University, Shanghai 200032, China

**Keywords:** histone deacetylase 1 (HDAC1), TCF-12, migration, invasion, GBC

## Abstract

The identification of prognostic markers for gallbladder cancer is needed for clinical practice. Histone deacetylases (HDACs) play an important role in tumor development and progression by modifying histone and non-histone proteins. However, the expression of HDAC1 in patients with gallbladder cancer is still unknown. Here, we reported that HDAC1 expression was elevated in cancerous tissue and correlated with lymph node metastasis and poorer overall survival in patients with GBC. Knockdown of HDAC1 using lentivirus delivery of HDAC1-specific shRNA abrogated the migration and invasion of GBC cells *in vitro*. TCF-12, as the HDAC1 binding protein, has also correlates with poor prognosis in GBC patients. And there is a positive correlation between HDAC1 and TCF-12 which leading the high invasion and migration ability of GBC cells. Taken together, our data suggested that HDAC1 and TCF-12 are a potential prognostic maker and may be a molecular target for inhibiting invasion and metastasis in GBC.

## INTRODUCTION

Gallbladder cancer, is a highly fatal disease with poor prognosis, has a mean 5-year survival rate of 5%, all stages combined. It is the most common malignant lesion of the biliary tract and the fifth most common among malignant neoplasms of the digestive tract [1]. Even with the numerous diagnostic tests available, gallbladder cancer is frequently first diagnosed during laparotomy or laparoscopy procedures, and the prognosis depends mainly on tumor stage at the time of diagnosis[2]. The aetiology of this tumour is complex, but there is a strong association with gallstones. Owing to its non-specific symptoms, gallbladder carcinoma is generally diagnosed late in the disease course [3, 4]. The survival rate of patients with Gallbladder cancer (GBC) treated with conventional methods remains very low due to poor prognosis. Even when combining radiation therapy and concurrent chemotherapy drug gemcitabine and cisplatin, the median patient survival is limited to very low level [5, 6]. Therefore, the identification of genes for effective targeting of GBC is a priority in current research related to the treatment of this type of tumor.

A wide range of genetic and epigenetic modifications have been shown to play a pivotal role in the development and tumorigenesis of cancer [7, 8]. These epigenetic changes are associated with DNA methylation and histone modifications. Understanding epigenetic changes may help to identify a novel cancer related network that may represent attractive targets for GBC treatment and provide new insights into the biological characteristics of GBC[9–11]. Histone deacetylases (HDACs) remove acetyl groups from histones, resulting in chromatin compaction and decreased accessibility to DNA for interacting molecules such as transcription factors, resulting in compaction of chromatin structure and transcriptional repression. HDACs operate by direct association with DNA-binding factors and by incorporation into large multifunctional repressor complexes such as Sin3, NuRD, and PRC2 [12]. In addition to functions in chromatin remodeling, HDACs deacetylate certain transcription factors, such as P53 [13], resulting in their decreased activity. HDACs form a large family, of which class I HDACs, including the closely related proteins HDAC1 and HDAC2, show the strongest histone deacetylase activity[14]. Of these, histone deacetylase 1 (HDAC1) was the first mammalian protein identified to have histone-directed deacetylase activity. HDAC1 plays important roles in regulating the cell cycle and is required in the transcriptional repression of cell-cycle genes such as P21/WAF [15], E2F1, and cyclins A and E [16]. The association of HDAC1 with promoter regions of specific genes is linked to their transcriptional repression. Nevertheless, it remains unclear which genes in particular are regulated by HDAC1 during invasion and migration, and the mechanism of how HDAC1 regulates invasion requires further investigation.

Basic helix-loop-helix (bHLH) proteins are transcription factors belonging to a large multigene family with important roles in animal development, including myogenesis, neurogenesis, hematopoiesis, and gut development [17–19]. They form homo- or heterodimers through the HLH motif and, consequently, bind to specific DNA sequences such as E-box (CANNTG) or N-box (CACGCG or CACGAG) through the DNA-binding basic domains. Transcription factor 12 (TCF12) is believed to be a transcription factor. It is a member of the basic helix-loop-helix family of proteins that recognizes the DNA E-box motif [20, 21]. It has been shown to form homo-oligomers or hetero-oligomers with myogenin, E12 and ITF2, and interacts with PTF-1 and RUNX1T1 [22]and the TCf12 regulator, ID1 [23, 24]. TCF12 has been shown to have a role in neuron differentiation and is upregulated in some tumors. All three of the genes we chose to study (Tcf12, Ccnd1 and Pctk1) are abundantly expressed in hippocampal neurons. TCF-12 have been reported [25] close relationship with invasion of cancer.

In this study, we investigated the expression of HDAC1 in GBC tissues. Then, we tested the role of HDAC1 in inducing tumor cell invasion in GBC cells via genetic elimination inhibition. We have identified TCF12 as a specific target gene of HDAC1. Overexpression of HDAC1 induced TCF12 deacetylation of promoter, further leading to increase in cellular invasion and migration. Moreover, we found that TCF12 targets HDAC1 to downregulate EMT markers. Therefore, our studies reveal TCF12 by binding with HDAC1 to influence the invasion of GBC cells. Our results provided a novel insight that HDAC1 may serve as a prognostic indicator and potential molecular target in the pathogenesis of GBC.

## RESULTS

### High HDAC1 expression and prognosis in GBC

**Figure 1:**
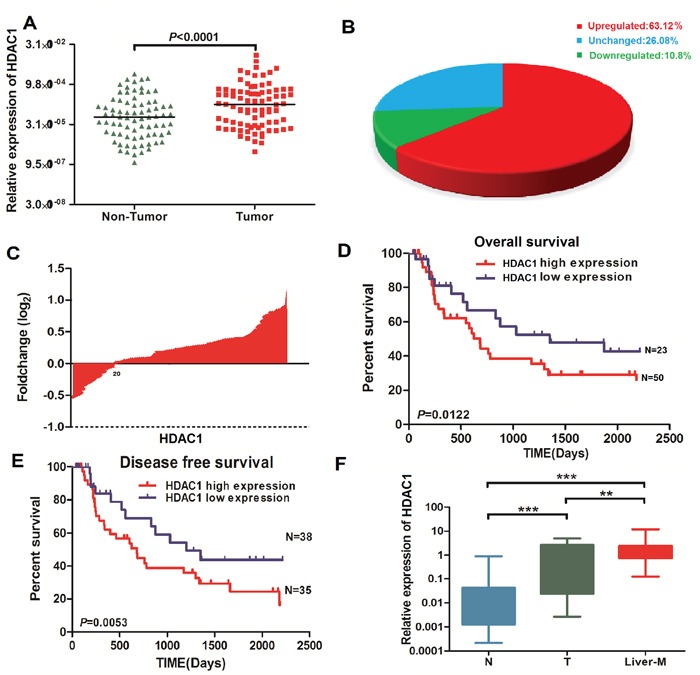
Overexpression of HDAC1 mRNA in GBC tissues and the relationship with overall survival in GBC patients **A.** and **B.** mRNA levels of HDAC1 were analyzed by Real-time PCR in 73 paired GBC tissue and normal tissue. **C.** The fold change of HDAC1 showed it has a high expression in GBC tissues. **D-E.** The overall survival and disease free survival of patients with High/low of HDAC1 of GBC patients. **F.** The expression level of HDAC1 in normal, GBC tissue, and liver-metastases group. ***P < 0.001, compared with normal tissues.

**Figure 2:**
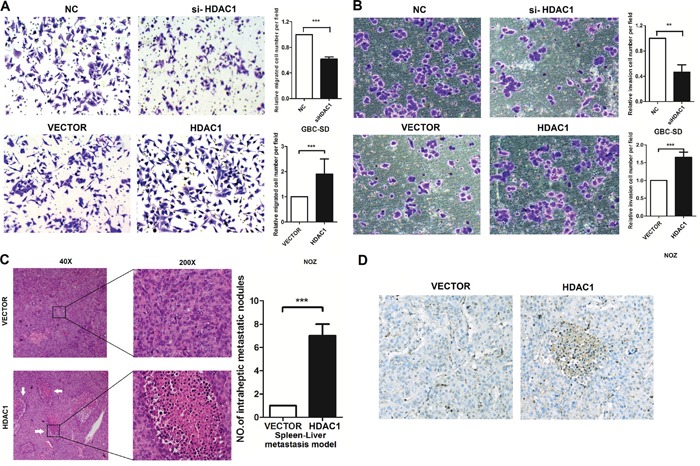
HDAC1 regulates cell invasion in GBC cancer cells **A.** and **B.** Transwell assay showed that knockdown of HDAC1 inhibited the invasion of GBC cell lines. Overexpression of HDAC1 restored the phenomenon. **C.** and **D.** *In vivo*, Immunohistochemistry showed that overexpression of HDAC1 promoted the Spleen-Liver-Metastasis. And HDAC1 have a significantly overexpression in metastatic sites. ***P < 0.001.

At first, HDAC1 mRNA and protein levels were compared in 73 paired GBC cancer tissues and normal gall bladder tissues. We found that HDAC1 mRNA expression was significantly up-regulated in cancer tissues (also confirmed in Supplementary Figure 1A). Positive HDAC1 expression was occupied 63.12% GBC samples (Figure 1A–1C, P < 0.0001), indicating that increased HDAC1 expression is frequent events in GBC. Besides, we evaluated the relationship between the HDAC1 expression and overall survival after surgery in patients with GBC. The results were similar in both cohorts between micro-assay results and tissue IHC staining (Figures 2A and 2C). The GBC patients with lower HDAC1 expression were associated with a survival benefit, suggesting HDAC1 expression was significantly associated with the prognosis of patients with GBC. According to Kaplan–Meier curves, the log-rank analysis indicated that patients with a higher expression of HDAC1 were significantly inferior to overall survival as compared to GBC patients with lower expression of HDAC1 (p < 0.05, Figure 1D and 1E). Figure 1F showed HDAC1 have an increase expression level in GBC tissue and Liver-metastasis tissue contrast with Normal gall bladder tissues. A summary of clinical information for these patients is shown in online Supplementary Table 1.

### The biological functions of HDAC1 *in vitro* and *in vivo*

To evaluate the oncogenic properties and effects of HDAC1 on GBC, we established GBC cell lines with HDAC1 stable overexpression or knockdown (Supplementary Figure 1). Knockdown HDAC1 expression in GBC-SD and NOZ cells leads to a significant decrease in invasion. On the other hand, HDAC1 overexpression in GBC-SD and NOZ cells leads to increase in invasion. As shown in Figure 2A–2B, silencing HDAC1 significantly decreased invasion of GBC cells, and the invasion ability of overexpression of HDAC1 was increased. And we checked the proliferation of HDAC1 in GBC cells, showed in Supplementary Figure 2, have little function on proliferation and colony formation.

*In vivo*, showed in Figure 2C–2D, to evaluate the function of HDAC1 in GBC development, we analyzed HDAC1 level and its distribution in GBC tissues by immunohistochemical (IHC) staining. HDAC1 levels in the tissues were assessed based on the staining density scores. We found that, when HDAC1 has a high level in vivo, the more metastatic lymph nodes occurred in spleen-Liver metastasis model. Statistical analysis revealed that HDAC1 level significantly increased in cancer tissues and it promotes the invasion of GBC cells in vivo and in vitro.

### Identifying TCF-12 as a HDAC1-interacting protein

**Figure 3:**
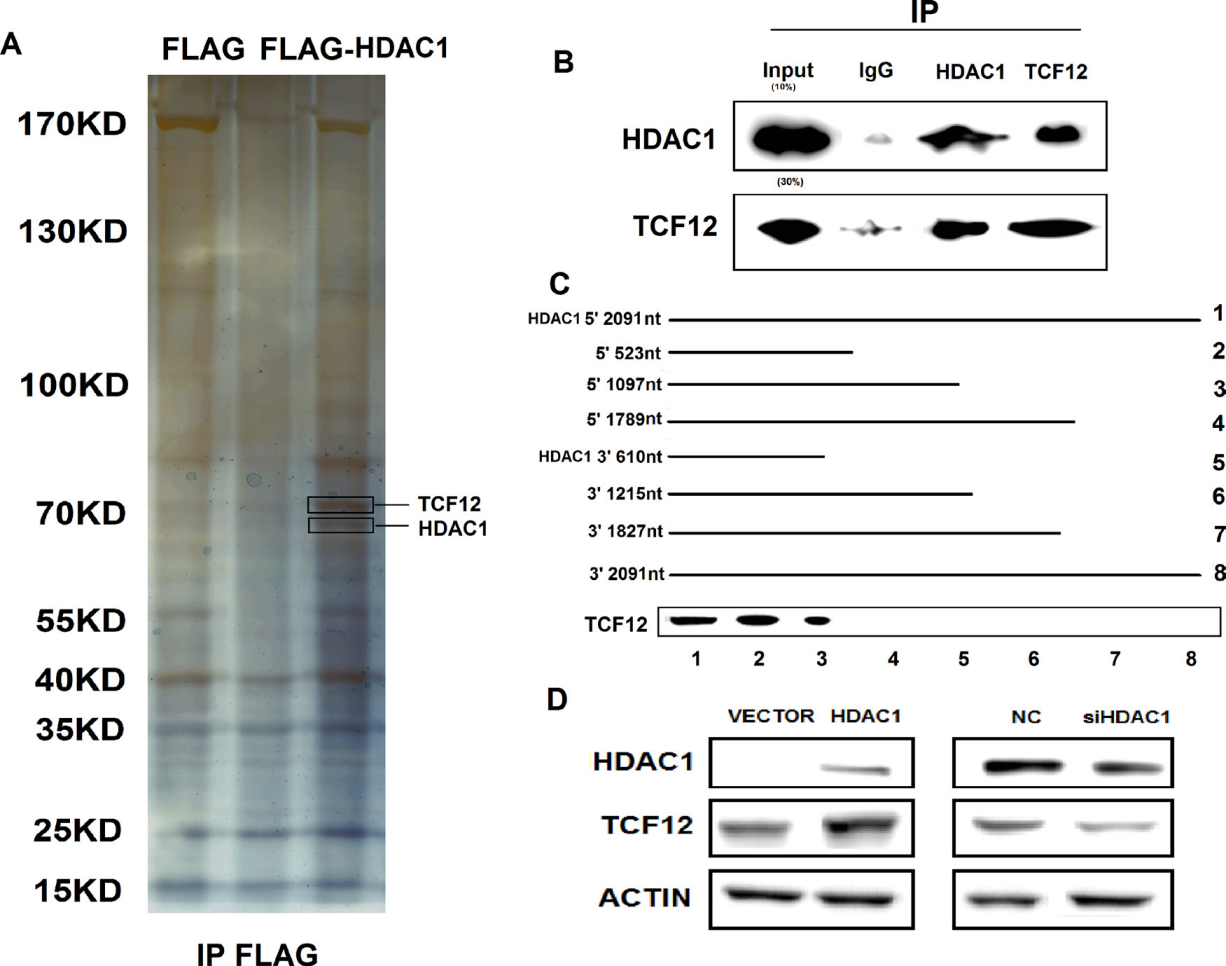
Immunoprecipitation of Flag-HDAC1 and the mechanism between HDAC1 and TCF-12 **A.** SDS-PAGE for immunoprecipitation of HDAC1 with antibody of Flag. **B.** Co-immunoprecipitation showed that HDAC1 and TCF-12 are binding together. **C.** TCF12 corresponding to different fragments of HDAC1 was biotinylated and incubated with GBC-SD whole cell extracts, captured with streptavidin beads and washed. Associated TCF-12 protein was detected by western blot. **D.** When knockdown of HDAC1, the expression level of TCF-12 was also decreased; and when overexpression of HDAC1, the level of TCF-12 has increased either.

**Figure 4:**
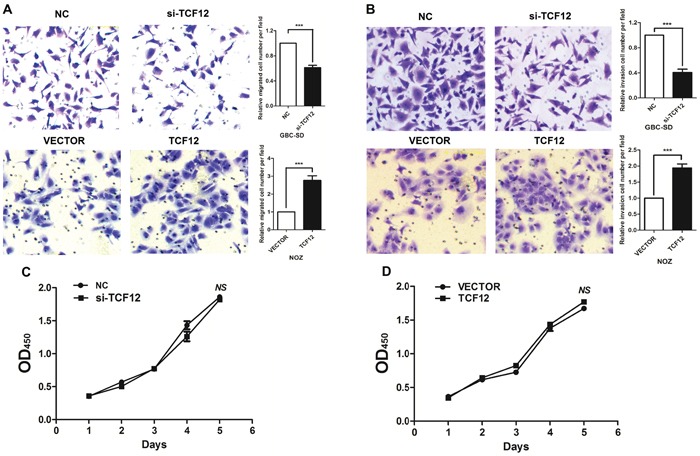
TCF-12 as an oncogene in GBC cell lines Transwell assay showed that knockdown of TCF-12 inhibited the invasion of GBC cell lines. Overexpression of TCF-12 increase the invasion of GBC cells. CCK-8 assay showed TCF-12 have little effect on proliferation of GBC cells. Knockdown of TCF-12 inhibited the invasion ability of GBC cells. Knockdown TCF-12 in high-expression of HDAC1 cells, the invasion ability of GBC cells has increased.

HDAC1 regulate molecular pathways via their interactions with proteins. To identify the binding proteins are associated with HDAC1 using an Immunoprecipitation assay. Among all of the proteins identified by mass spectrometry, only TCF-12 was detected by western blotting from three independent Immunoprecipitation assays (Figure 3A and Supplementary Excel 1). We further performed Co-IP with an antibody against TCF-12 and HDAC1 using proteins from the GBC-SD cells. We observed HDAC1 enrichment using the TCF-12 antibody contrast with a non-specific antibody (IgG control) (Figure 3B). To finding the binding site of TFC-12, we performed deletion mapping analyses identified a 523-nt region at the 5′ end of HDAC1 that is required for its association with TCF-12 (Figure 3C). Together, these results demonstrate a specific association between HDAC1 and TCF-12. We sought to determine the functional relevance of the association between HDAC1 and TCF-12. We detected the expression level of TCF-12 in HDAC1 knockdown and overexpression in GBC-SD cells. Results showed a reduction inTCF-12 in si-HDAC1 cells in protein levels, otherwise, overexpression of HDAC1, the expression level of TCF-12 was increased (Figure 3D). These findings reveal that, TCF-12 was binding with HDAC1 and there is a positive relationship between them. And in Figure 4, we evaluate the function of TCF-12 *in vitro*, knockdown of TCF-12 made decrease of GBC-SD cells invasion. Overexpression of TCF-12 lead increase of NOZ cells invasion. But, TCF-12 have little effect on proliferation of GBC cells. The results reveal TCF-12 as an oncogene in GBC cancer cells.

### Effect of HDAC1 on expression of epithelial and mesenchymal markers

**Figure 5:**
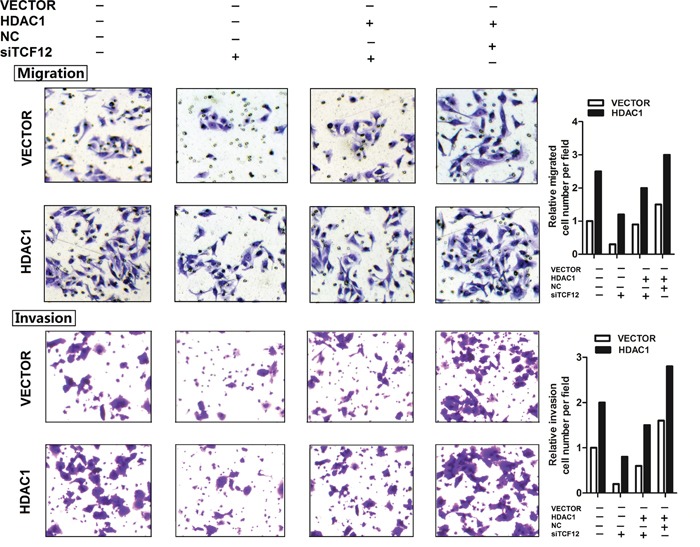
TCF-12 as an oncogene in GBC cell lines Transwell assay showed that knockdown of TCF-12 inhibited the invasion of GBC cell lines. Overexpression of TCF-12 increase the invasion of GBC cells. CCK-8 assay showed TCF-12 have little effect on proliferation of GBC cells. Knockdown of TCF-12 inhibited the invasion ability of GBC cells. Knockdown TCF-12 in high-expression of HDAC1 cells, the invasion ability of GBC cells has increased.

From above results, we studied the function of HDAC1 and TCF-12 in migration and invasion of GBC cells. Figure 5 showed, si-TCF12 in GBC-SD cells, the migration and invasion were decreased significantly. When si-TFC12 in stable overexpression HDAC1 cells, the invasion and migration ability were also decreased contrast with the un-treated group, but the cell numbers were much more than the si-TCF12 group. And in stable overexpression HDAC1 cells, the cells have the super ability of invasion and migration. These results illustrated TCF-12 as an oncogene in GBC invasion and migration, and there is a positive relation between HDAC1 and TCF-12 can promote the invasion of GBC cancer cells.

**Figure 6:**
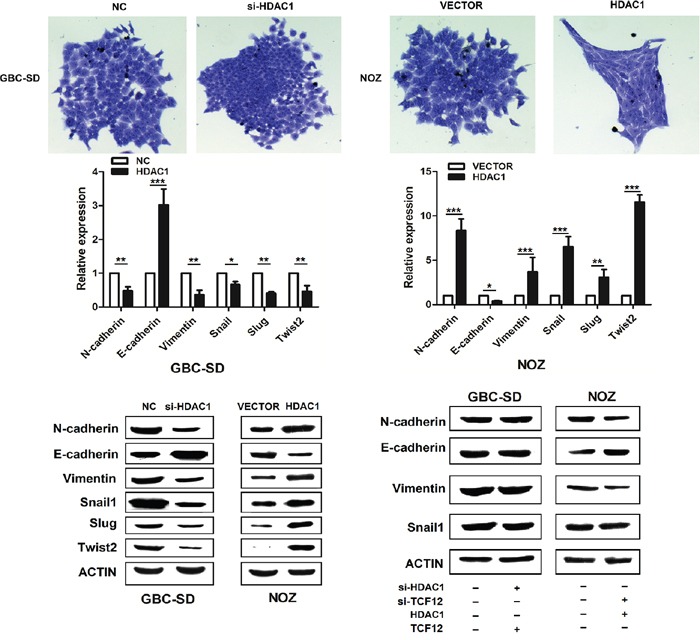
HDAC1 has promote EMT progress The cellular morphology of si-HDAC1 and overexpression of HDAC1 group have changed significantly. Overexpression of HDAC1, the morphology of NOZ has become thin and long spindle. The expression level of mRNA and protein of EMT markers have changed along with the HDAC1.

In the colony assay, we observed in overexpression HDAC1 NOZ cells, the phenocopy changes with epithelial- mesenchymal transition (Figure 6). So, we detected the EMT markers in mRNA and protein levels. We found that when overexpression HDAC1 in NOZ cells, the expression of the epithelial markers E-Cadherin decreased, and the mesenchymal markers such as N-cadherin, Vimentin, Snail1, Slug and Twist2 were increased significantly. On other hand, knockdown HDAC1 in GBC-SD cells, we gain the opposite results. These findings reveal HDAC1 play a fatal role in invasion by influenced the EMT markers. To explored the function of TCF-12 in this process, we overexpression TFC-12 in si-HDAC1 GBC-SD cells, we did not observe the epithelial markers E-Cadherin reduction in protein levels. Then, we check the TCF-12 target genes TWIST1, TWIST 2, NOTCH1, HAND1 and MYOD1, showed HDAC1 and TCF-12 deregulated EMT progress by these genes (Supplementary Figure 3). Also, we knockdown TCF-12 in overexpression HDAC1 NOZ cells, the protein level of mesenchymal markers have no change.

### Positive expression of HDAC1 and TCF-12 correlates with poor prognosis in GBC patients

We hypothesised that TCF-12 might act as an oncogene, and if so, TCF-12 overexpression should be a frequent event in GBC. Therefore, we used qRT-PCR to determine the TCF-12 expression levels in tumor and paired non-tumour tissues obtained from 73 patients with GBC.

**Figure 7:**
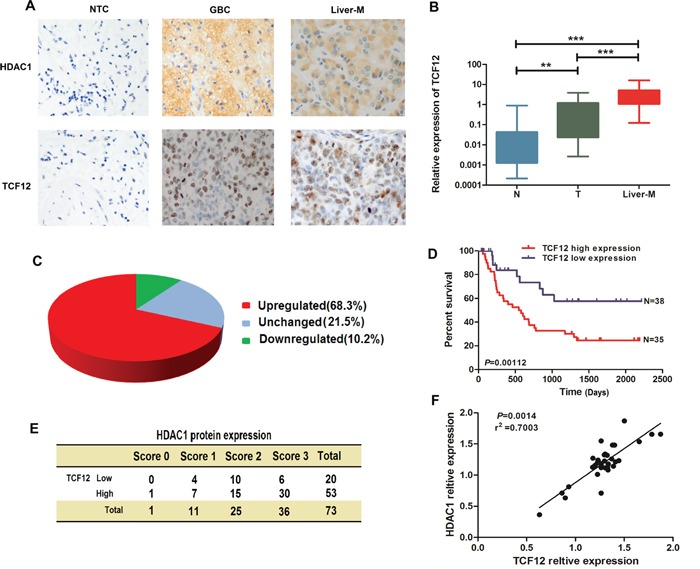
HDAC1 and TFC-12 have a co-expression and TCF-12 has closely related to the prognosis of GBC **A.** HDAC1 and TCF-12 expression was investigated by immunohistochemistry (IHC) in normal tissue, GBC tissue, and Liver-Metastasis tissues. **B-C.** There were significant differences of HDAC1 expression between cancerous and non-cancer tissues (**P< 0.05, ***P< 0.001). **D-E.** Kaplan–Meier curves showed the survival benefit in GBC patients with low TCF-12 expression relative to that in GBC patients with high TCF-12 expression. **F.** Positive correlation between HDAC1 and TCF-12 in 30 paired GBC tissues.

As shown in Figure 7A, both HDAC1 and TCF-12 have a high-expression in GBC and Liver-metastasis tissues contrast with Non-tumor tissues. Then, we analyzed the mRNA level of TCF-12 in normal, GBC, and Liver-metastasis tissues (Figure 7B–7C). We found TCF-12 has a high-expression in GBC and Liver-metastasis tissues (68.3% of total patients) with P<0.0001. In a multivariate Cox regression model, high TCF-12 expression levels in tumors were associated with a poor survival prognosis (Figure 7D). We made a correlation analysis in 73 paired GBC tissues, Figure 7E–7F, HDAC1 and TCF-12 have a positive correlation in GBC tissues. Taken together, these results suggested that HDAC1 and TCF-12 overexpression plays a role in GBC carcinogenesis.

## DISCUSSION

Metastasis is a highly organ-specific process, which requires multiple steps and interactions between tumor cells and the host. These include detachment of tumor cells from the primary tumor, intravasation into lymph and blood vessels, survival in the circulation, extravasation into target organs, and subsequent proliferation and induction of angiogenesis[26, 27]. In recent years, the development of molecular biology has led to the successful exploration and identification of biomarkers. To date, a large number of biomarkers have been proposed for GBC progression and aggressiveness [28]. Regulation of both coding genes and noncoding RNAs in GBC has been suggested to have considerable potential for predicting the diagnosis and prognosis of patients with GBC[29].

In this study, we compared the gene profiles of GBC, normal gall bladder tissues using the Arraystar Gene Expression Microarray. The gene, HDAC1, displayed a remarkable trend of increased expression levels with the progression from normal gall bladder tissues to GBC. We validated the HDAC1 expression patterns in the GBC clinical samples. In addition, we showed that HDAC1 expression was associated with patient survival and predicted the response to adjuvant chemotherapy. Our findings suggest that HDAC1 plays an important role during GBC tumorigenesis. In this regard, our data contribute to a growing body of literature supporting the importance of oncogene in the field of GBC cancer research.

In the current study, we demonstrated an association between HDAC1 expression levels and CRC prognosis or therapeutic outcome. A robust association of high HDAC1 expression in tumors with poor survival was confirmed in 73 GBC samples. The association was independent of other clinical covariates, indicating that HDAC1 expression may be a useful prognostic biomarker to help identify patients at a higher risk of GBC progression. In addition, the patients whose tumors had increased HDAC1 expression had a poor response to adjuvant chemotherapy. These results indicate that HDAC1 status in tumors may be a useful tool for estimating prognosis of a patient with GBC and for selecting patients who are likely to benefit from adjuvant chemotherapy to prevent relapse.

We investigated the mechanisms by which HDAC1 exerts its function and modulates malignant GBC phenotypes in vitro and in vivo. Our data clearly indicated that silencing HDAC1 expression inhibited GBC cell migration and invasion in vitro and in vivo. The HDAC1L transcript was found to be associated with TCF-12 to promote invasion and migration of GBC. We also found that the ability of HDAC1 to enhance invasion and migration is in large part attributed to its ability to binding with TCF-12 and subsequent activation of the EMT signalling pathway. These findings provide additional evidence that HDAC1 and TCF-12 plays an important role in GBC tumorigenesis and progression.

Chromatin with histone tailing is defined as a critical regulator of gene transcription. Histone acetylation and deacetylation, catalyzed by multisubunit complexes, play a key role in the regulation of eukaryotic gene expression [30, 31]. The protein encoded by this gene belongs to the histone deacetylase/acuc/apha family and is a component of the histone deacetylase complex. HDAC1 activity is regulated by its binding to corepressor complex partners and depends on its posttranslational modifications. In particular, acetylation and phosphorylation of HDAC1 can modulate its enzymatic activity and complex formation, whereas sumoylation and ubiquitination regulate its stability.

Recent studies have demonstrated the functional roles of HDAC1 and provided insights into the molecular mechanisms by which Histone acetylation function in a variety of human tumors. However, the mechanisms regulating HDAC1 expression in GBC have not been thoroughly elucidated. Whether the translation factors can regulate the expression of HDAC1 remains largely unknown. Our results revealed that TCF-12 play a critical role in the regulation of HDAC1 expression in GBC. Our results, highlight the histone deacetylase1 and TCF-12 relationship between epigenetic regulation of HDAC1 and provide a novel sight for GBC study in the future.

To our knowledge, this is the first study reporting the effects of HDAC1 in GBC, which highlights the association of HDAC1 and TCF-12 in GBC cancer and opens up a new field for GBC treatment. Taken together, our results indicate that HDAC1 and TCF-12 were an oncogene that promotes the tumorigenesis and progression of GBC. This finding suggests that HDAC1 may be important targets for tumor therapy.

In conclusion, in the present study, we demonstrated that HDAC1 and TCF-12 expression were high in tumor samples from patients with in HCC. It is clear from the present study that the positive expression of HDAC1 and TCF-12 reflect the aggressiveness and poor prognosis of GBC, making their assessment potentially useful in clinical practice. In addition, a multivariate analysis revealed that HDAC1 and TCF-12 expression was an independent and significant risk factor for recurrence and survival, suggesting that this combination has prognostic value. In the era of personalized medicine, the identification of patients who are at a high risk of early recurrence may provide clinicians with opportunities for early interventions and improve the outcomes of HCC. The findings of the current study may help to determine optimal treatment strategies.

## MATERIALS AND METHODS

### Cell culture, antibodies and patients

Human GBC cells GBC-SD and NOZ, HEK293T, were obtained from the American Type Culture Collection. Cells were maintained in Dulbecco's Modified Eagles Medium (DMEM) (both from Invitrogen Life Technologies, Carlsbad, CA, USA) supplemented with 10% fetal bovine serum (FBS) (both from Invitrogen Life Technologies, Carlsbad, CA, USA) and 1% antibiotics conditions (5% CO2) at 37°C.

The following antibodies were used: Rabbit anti-human HDAC1 antibody and rabbit anti-human TCF-12 antibody (CST, CA, USA); antibodies of EMT markers which include: E-cadherin, N-cadherin, Vimentin, Snail1, Snail2(slug) Twist(CST, CA, USA); normal mouse or rabbit IgG (Santa Cruz Biotechnology, Santa Cruz, CA, USA); mouse anti-FLAG, mouse anti-GFP (Sigma, CA, USA).

We included 73 cases of GBC cancer patients, from Feb 2013 to Nov 2015, who underwent primary tumor resection at Zhongshan Hospital affiliated with the Fudan University (Shanghai, China). In our study, all patients did not receive chemotherapy or radiotherapy before surgery. The clinico-pathological information and patients’ medical history were documented during post-operative follow-up. Prior to our scientific research, patient's consent was obtained from the Institute Research Ethics Committee of the Fudan University.

### Immunohistochemistry and evaluation of immunohistochemistry staining

All sections were dewaxed in xylene and rehydrated in graded ethanol, followed by incubating in 3% hydrogen peroxide for 10 min to quench endogenous peroxides. Samples were heated in 0.01 mol/L citrate buffer for 15 min at 100°C, and then put at room temperature for 30 min. After cooled, samples were blocked with 2% normal goat serum in PBS for 30 min to block antigenic epitopes, and then incubated with primary antibody (1:500 dilution) at 4°C overnight. After that, the sections were washed with PBS for three times, and then incubated with system-labeled HRP anti-mouse secondary antibody at room temperature for 20 min. Next, the sections were incubated in DAB and counterstained in Mayer's hematoxylin, dehydrated in alcohol and xylene. PBS was used as negative control. Under the microscope, the positive areas seem like brown yellow granules. The score of the immunohistochemistry staining was evaluated by one investigator who was blinded to this study. And the sections were scored based on the positive percentage and staining intensity. Sections were defined as positive if there were brown yellow platy or granules in the plasma of the cells. The intensity of plasma staining was scored and graded by four scales: 0 (0% cells); 1 (0–25% cells); 2 (25–50% cells); and 3 (>50% cells).

### siRNA, RNA extraction and real-time PCR

Cells were seeded into 6-well plates and then transfected with 10 nM siGENOME non-targeting siRNA, human HDAC1 or TCF-12 (Ribobio, China). Total RNAs were isolated from tissues or cells by TRIzol reagent (Invitrogen, USA), and reverse transcriptions were performed by Takara RNA PCR kit (Takara, Japan), following the manufacturer's instructions. In order to determine the transcripts of the interest genes, Real-time PCR was performed using a SYBR Green Premix Ex Taq (Takara, Japan). β-actin gene was used as an internal control. The primers used were as following: HDAC1 (Forward: 5′-CATCTCCTCAGCATTGGCTT-3′; Reverse: 5′-TATTATGGACAAGGCCACCC-3′); E-cadherin (Forward: 5′-GGCTCCTGGCAAAAGGTCA-3′; Reverse: 50-CTGCGTAGTTGTGCTGATGT-3′); N-cadherin, (Forward: 5′-TGGAGCCCGTG AAAAAGAGC-3′; Reverse: 5′-TCTCCTTCATCTTAGAGGCCAC-3′); Vimentin (Forward: 50-ACCTTCCGCAGTGCTCCTA-3′; Reverse: 5′-CCCAGCCAAGAAACGGTCC-3′); Snail1 (Forward: 5′-ACTCAACGTGCAAGCCTCG-3′; Reverse: 5′-GCTCAAGAAAGTGCTGATCCC-3′); Snail2(slug) (Forward: 5′-GTGAAGGCGCTATTTGGCG-3′; Reverse: 5′-TGGTTG CTCATAATCACTGCC-3′); Twist (Forward: 5′-CGAAGGTCAAGCTATGAGGACA-3′; Reverse: 5′-ATCTGCGATGCTGGCAATCT-3′). TCF-12 (Forward: 5′-GCTCGACGCTAGGATCTGAC-3′; Reverse: 5′-GCTTTCCACGACGGTGAC-3′); β-actin (Forward: 5′-CATGTACGTTGCTATCCAGGC-3′; Reverse: 5′-CTCCTTAATGTCACGCACGAT-3′). All PCR reactions were performed with a 7900 Fast Real-Time PCR system (Applied Biosystem).

### Invasion assay

Cell invasiveness was evaluated using a Transwell chamber assay (Costar, USA). Chamber membranes (8μm, BD Falcon) were pre-coated with 6μl matrigel at 4C overnight, and seeded with 1×10^5^ cells. DMEM without FBS supplement was added to the upper chamber and 600μl of DMEM (containing 10% FBS) was added to the lower chamber. Cells were incubated for 48 h with or without treatment. The cells on the top of membranes were removed, and the cells that penetrated the membrane were fixed in ethanol, followed by crystal violet staining. The number of cells on the opposite side of the membrane was counted under the microscope in four random fields of vision.

### Western blotting

Cells were lysed in a lysis buffer. Protein concentration was determined using the BCA Protein Assay kit (Pierce). Proteins (30μg) were subjected to 10%–12% SDS polyacrylamide gel electrophoresis and subsequently transferred onto Hybond ECL membranes (Amersham). After washing with 0.1% TBS-T, membranes were incubated for 1 h at room temperature in blocking buffer (5% skimmed milk in TBS-T) and then incubated with appropriate antibodies (1:500 dilution; rabbit anti-human HDAC1, rabbit anti-human TCF-12 antibodies are from Cell Signaling, Danver, MA; others are from Santa Cruz Biotechnology, Santa Cruz, CA) overnight at 4°C. After washing with TBS-T, membranes were reacted with horseradish-peroxidase-conjugated anti-rabbit antibody (1:2000 dilution, Santa Cruz) for 2 h at room temperature. After further washing with TBS-T, immunoreactive proteins were detected using the Super Signal West Pico Chemiluminescent Substrate (Pierce, Woburn, MA). The efficiency of siRNA and overexpression of HDAC1 and TCF-12 by western-blot also provided in supplementary Figure 1C.

### Colony formation assay

For colony formation assays, 1500 cells were plated in 6-wellplates. The culture medium was changed twice per week. Fourteen-days after plating, cells were fixed in 4% formaldehyde and stained with crystal violet. Colonies larger than 1 mm (>50 cells/clone) in diameter were counted. The results were presented as colony forming rate (colony forming numbers per 100 plated cells).

### Lentivirus-mediated shRNA knockdown of HDAC1 in GBC cells

Pre-synthesized shRNAs against human HDAC1 were synthesized and tested. The constructed transfer vectors, Packaging plasmid, envelope plasmid were co-transfected into HEK293T cells at a molar ratio of 4:3:1.2μg/μl and the culture supernatant contained the viral particles was harvested 48 h after transfection and clarified with a 0.45 μm membrane filter (BD Biosciences, CA, USA). The supernatant was then condensed by PEG-8000 (1:4) resuspended in the cell culture medium, and stored at −80°C. The medium with the viral particles was used directly to infect GBC-SD and NOZ cells. A lentivirus preparation that carried a non-target “non-sense” shRNA (scrambled shRNA) was used as the control.

### Immunoprecipitation

Confluent cells in 60-mm dishes were washed with PBS and cells were removed using a cell scraper and centrifuged at 2500g for 5 min at 4°C. Lysate was prepared using a modified radioimmune precipitation assay (mRIPA) buffer (50 mM Tris, pH 7.5, 150 mM NaCl, 5 mM EDTA, 0.1% Triton-X100, 0.1% Nonidet P-40, and 10 μg/ml each of leupeptin, aprotinin, and 4-(2-aminoethyl) benzenesulfonyl fluoride). The sample was added with 1μg anti-HDAC1 antibodies by overnight incubations at. Protein-A agarose beads (30μl) (Santa Cruz Biotechnology, USA) were added to the lysate, and the mixture was incubated under shaking at 4°C for 1 h. The beads were collected using centrifugation and washed three times with mRIPA buffer. Proteins binding to the beads were eluted by adding 2 x sample treatment buffer boiling in 100°C for 10 mins for western-blot.

### Tumor model in nude mice

Age-matched adult male nude mice, four weeks old, were housed in a temperature- and light-controlled environment with a 14/10-h light/dark cycle. The GBC-SD cells were harvested in serum-free culture medium and the concentration of the cell suspension was adjusted to 1×10^7^ viable cells per ml. The cell suspension (0.2 ml) was injected in the liver. The tumor was formed within approximately 18 days after injection. After 30 days of observation, the mice were sacrificed and an autopsy was immediately performed. The tumor tissues were then fixed in 4% paraformaldehyde.

### Statistical analysis

The ANOVA test was used to assess the significance of the differences among the experimental groups. The results are represented as means± standard deviation (S.D.). All the cellular experiments were performed three or four times. Data are presented as mean ± SEM. Statistical differences were determined by a two-tailed t test. A Kaplan–Meier survival curve was drawn using Graphpad Prism 5 (Graphpad Software Company, USA), and the significance was calculated with the log-rank value. Statistical significance is displayed as *(P < 0.05), ** (P < 0.01) or *** (P < 0.001).

## SUPPLEMENTARY FIGURES AND TABLES



## Abbreviations

HDAC1: Histone deacetylases 1
GBC: Gallbladder cancer
TCF-12: transcription factor 12
IHC: immunohistochemical
Liver-M: spleen-Liver metastasis
EMT: Epithelial- Mesenchymal Transition.
