# Supplementary material for: HDAC1 promoted migration and invasion binding with TCF12 by promoting EMT progress in gallbladder cancer
Source: Oncotarget. 2016 Apr 15;7(22):32754–64. doi: 10.18632/oncotarget.8740 (PMC5078048; doi:10.18632/oncotarget.8740)
Supplement: Supplementary file 1 [file oncotarget-07-32754-s001.pdf]

HDAC1 promoted migration and invasion binding with TCF12 by promoting EMT progress in gallbladder cancer

SUPPLEMENTARY FIGURES AND TABLES

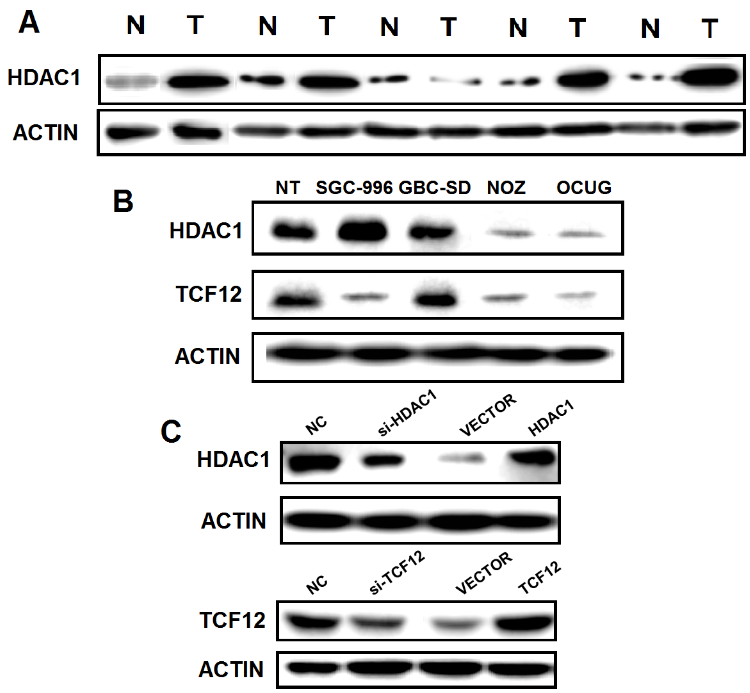

Supplementary Figure S1: A. the expression level of HDAC1 in normal gall bladder tissue and GBC tissues. **B.** The expression level of HDAC1 in different GBC cell lines. **C.** The efficiency of siRNA of HDAC1 and TCF-12 by western blot, and the stable overexpression of HDAC1 and TCF-12 by western blot.

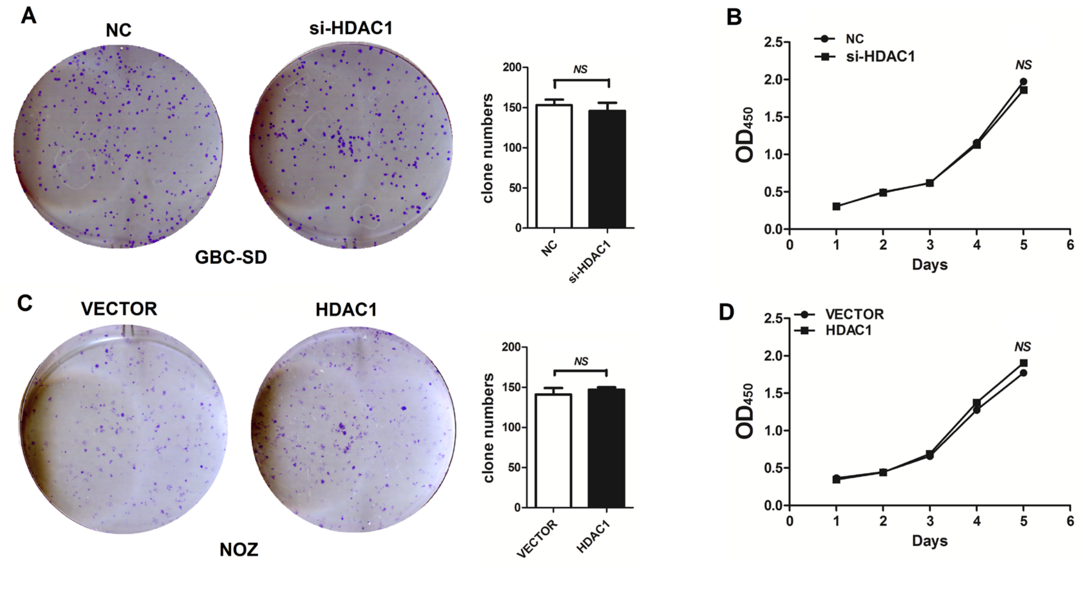

Supplementary Figure S2: HDAC1 has little effect on GBC cell proliferation. A-B. Colony formation assay of si-HDAC1 and overexpression of HDAC1 in GBC cell lines; **C-D.** CCK-8 assay showed HDAC1 have little effect on proliferation of GBC cells.

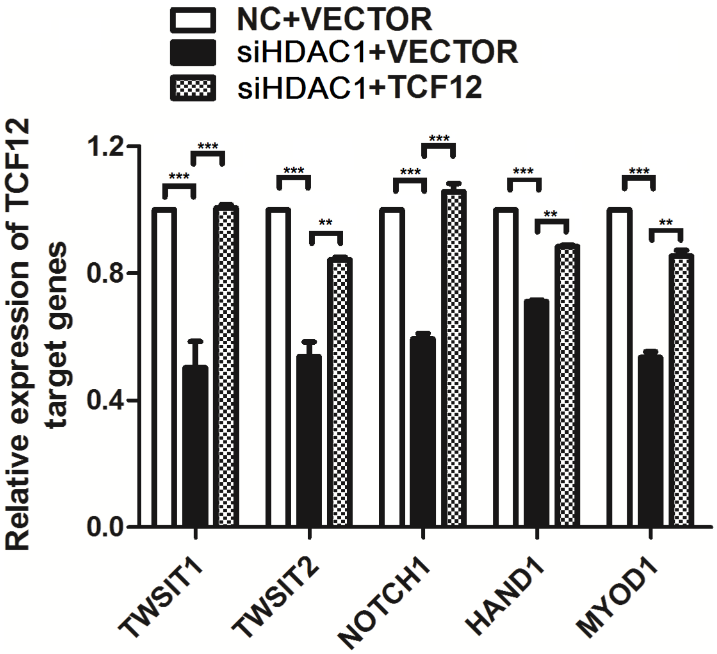

Supplementary Figure S3: the expression of TCF-12 target genes testing by Q-PCR.

Supplementary Table S1: Relationship between HDAC1 expression and clinicopathologic factors of patients with Gallbladder Cancer

Parameter	**No.** of patients	HDAC1(low)	HDAC1 (high)	*P* -value
Sex				0.3506
male	76	32	44	
female	25	12	13	
Age (yr)				0.1123
< 60	68	30	38	
≥ 60	33	20	13	
Tumor differentiation				**0.0001**
I	10	3	7	
II	65	15	50	
III	35	5	30	
Tumor size (cm)				0.2011
≤5	55	34	21	
>5	50	28	22	
Differentiation grade				0.41254
Well-moderate	54	23	31	
Poor-undifferentiation	47	19	28	
T stage				**0.0213**
T1-T3	57	16	41	
T4	48	5	11	
Lymph node status				
Negative	32	10	22	**0.00170**
Positive	73	12	61	
Distant metastasis				0.5210
M0	48	30	18	
M1	57	22	35	
TNM stage				0.0831
I-II	57	27	30	
III-IV	48	26	22	
Lymphatic invasion				**0.00702**
Negative	20	3	17	
Positive	85	24	61	
Venous invasion				0.0811
Negative	50	28	22	
Positive	55	35	20	

Supplementary Table S2: Univariate analysis identifies factors influencing the overall survival rate of Gallbladder Cancer patients

Factors	HR	95% CT	P value
Sex	0.985	1.01-2.41	0.631
Age(>60 vs.≤60)	1.521	0.824-1.8921	0.752
Tumor size(cm)>5	4.113	2.15-12.36	0.0589
Tumor size(cm)<5	2.117	0.98-2.86	0.0821
Differentiation grade	4.532	2.115-6.101	**0.0183**
T stage	1.245	0.6-3.11	**0.042**
Lymph node status	2.01	1.03-4.52	0.056
Distant metastasis	5.13	2.102-9.02	**0.0015**
TNM stage(IIIvs.IIvs.I)	0.921	0.364-2.117	0.23
Lymphatic invasion	3.2	1.10-6.58	**0.001**
Venous invasion	0.354	0.117-6.04	0.651
HDAC1 expression	3.66	1.08-7.223	**0.0021**

HR: hazard ratio; CI: confidence interval; TNM: tumor–node–metastasis classifications.

Supplementary Table S3: Multivariate analysis identifies factors influencing the overall survival rate of Gallbladder Cancer patients

Factors	HR	95% CT	P value
Tumor size(cm)>5 vs. ≤5	1.531	1.01-3.11	0.080
Differentiation grade	3.15	2.13-5.12	**0.0122**
T stage	1.02	0.36-3.20	**0.007**
Lymph node status	2.10	0.96-4.09	0.089
TNMstage(IIIvs.II vs.I)	1.75	1.02-2.69	**0.0013**
Lymphatic invasion	0.89	0.79-2.13	**0.0051**
Venous invasion	1.01	0.885-2.613	0.389
HDAC1 expression	3.11	2.05-4.56	**0.0018**

HR: hazard ratio; CI: confidence interval; TNM: tumor–node–metastasis classifications.
